# Comparative study of the vulnerability profile of young people and adults with reactive HIV tests*

**DOI:** 10.1590/1980-220X-REEUSP-2025-0119en

**Published:** 2025-10-13

**Authors:** Pedro Augusto Bossonario, Rubia Laine de Paula Andrade, Jaqueline Garcia de Almeida Ballestero, Gabriela Tavares Magnabosco, Gabriel Pavinati, Aline Aparecida Monroe

**Affiliations:** 1 Universidade Estadual de Maringá, Departamento de Enfermagem, Maringá, PR, Brazil.; 2 Universidade de São Paulo, Escola de Enfermagem de Ribeirão Preto, Departamento de Enfermagem Materno-Infantil e Saúde Pública, Ribeirão Preto, SP, Brazil.

**Keywords:** HIV, Health Profile, HIV Testing, Sexual Behavior

## Abstract

**Objective:**

To identify and compare the vulnerability profile of young people and adults with reactive tests for human immunodeficiency virus in a large Brazilian municipality.

**Method:**

Descriptive epidemiological study involving 84 young people and 147 adults who visited one of the municipality’s Testing and Counseling Centers between 2018 and 2021 and tested positive for human immunodeficiency virus. Data were collected using a structured self-administered questionnaire. Descriptive data analysis, chi-square test, Fisher’s exact test, and standardized and adjusted residual analysis were performed.

**Results:**

There was a statistically significant association between being young and single, having completed high school, being a student, being a man who has sex with men, and not having been tested for human immunodeficiency virus and syphilis at the time of diagnosis, as well as an association between adults who were married/in a stable relationship, had completed higher education, were employed, were heterosexual, and had partners with sexually transmitted infections.

**Conclusion:**

Understanding the vulnerability profile of people who test positive for human immunodeficiency virus helps guide public health actions aimed at controlling virus transmission among population groups that require greater attention.

## INTRODUCTION

Approximately 39 million people were living with human immunodeficiency virus (HIV) worldwide in 2022, of whom 29.8 million were receiving antiretroviral treatment, 1.3 million were newly diagnosed, 37.5 million were over 15 years of age, and 2.2 million resided in Latin America^(1,2)^. This context reinforces HIV as an important public health problem worldwide, placing it at the forefront of policies to eliminate the infection.

In Brazil, between 2007 and 2022, 469,357 new cases of HIV were reported in the Notifiable Diseases Information System (SINAN), with annual records exceeding 40,000 cases since 2015, except in 2020, during the COVID-19 pandemic^(3)^. In 2022, 43,403 new cases of HIV were registered, while between 2018 and 2021, the period corresponding to the present study, 169,383 people were reported with HIV, of whom 161,060 were between 15 and 59 years of age^(3)^.

Among young people and adults, behaviors that increase vulnerability to HIV infection are observed, such as the use of legal and/or illegal drugs, inconsistent condom use, multiple partners, gender inequality, and other conditions that increase vulnerability to the virus^(4,5,6)^.

Thus, knowing the profile of people who test positive for HIV allows for the identification of individuals in vulnerable situations, especially with regard to individual, collective, and contextual aspects that, together, increase susceptibility to infections and diseases^(7,8)^. This understanding contributes to the targeting of public health policies for the prevention and control of virus transmission, in addition to favoring the reach of vulnerable populations through strategies adapted to the realities of people and health services.

Historically, understanding the profile of people living with HIV has evolved in three distinct phases of the epidemic. The first was marked by the predominance of homosexual men with different levels of education, considered at the time to be “risk groups.” In the second phase, the concept of “risk behavior” was adopted, with an emphasis on people who used injectable drugs. The third phase, in turn, reached the concept of vulnerability, incorporating individual, social, and programmatic dimensions, as well as taking into account the impact on heterosexuals, women, people with low levels of education, and the spread of AIDS in rural areas^(9)^.

Regardless of any classifications adopted over time, it should be recognized that vulnerability to HIV has several facets structured by biological, behavioral, social, and structural factors, which are better understood when observing the global distribution of HIV cases, distributed mainly among sex workers, injecting drug users, gay men and men who have sex with men (MSM), transgender women, clients of sex workers, and sexual partners of key populations. These groups account for the majority of cases, while the remaining population accounts for about 30%^(1,10,11)^.

Finally, the state of São Paulo had 36,025 new cases of HIV registered in SINAN and 32,681 cases of AIDS included in the Mortality Information System (MIS) during the period from 2018 to 2022, as well as an AIDS detection rate of 13.7 people per 100,000 inhabitants in 2022^(3)^. Given this scenario, this study aimed to identify and compare the vulnerability profile of young people and adults who tested positive for human immunodeficiency virus in a large Brazilian municipality.

## METHOD

### Type of Study

This is a descriptive epidemiological study using secondary data. It should be noted that the recommendations of Strengthening the Reporting of Observational Studies in Epidemiology (STROBE) were followed in the preparation of this article.

### Setting

The five Testing and Counseling Centers (TCC) in a large municipality in São Paulo were considered.

### Population and Selection Criteria

The study population consists of young people and adolescents who sought one of the five TCCs in the municipality studied to undergo rapid or serological testing for HIV between 2018 and 2021. All individuals who tested positive for the infection were included in the study^(12,13)^. Thus, according to the Brazilian Ministry of Health and other sources, the population composed of adolescents and young adults corresponds to those aged between 15 and 24 years, a classification adopted in this study. For the adult population, individuals aged between 25 and 59 years were considered.

### Data Collection

Between January and June 2022, the study data were collected from a secondary source, consisting of the TCC Service Record. This instrument is self-completed by people who attend the health service and consists of 42 questions, divided into eight sections: search for service; identification (sociodemographic data); contact information; risk factors for hepatitis; tuberculosis (respiratory symptoms or contact); use of psychoactive substances; sexually transmitted infections (STIs); and sexual behavior.

Six other sections of the form (population sample, type of exposure, testing, conduct, return, and observations/closure) comprise 10 questions, which are filled out by health professionals according to the answers given by TCC users and the results of tests and actions taken after such results. Thus, the responses to the instrument are filled out in an open or dichotomous manner and by multiple choice.

For data collection, all forms available at the service for people who had reactive tests during the stipulated year were considered and transcribed using an instrument developed using software called Research Electronic Data Capture (REDCap).

### Data Analysis and Processing

In the present study, the profiles of young people and adults were compared according to sociodemographic variables (gender identity, race/color, marital status, education, work, alcohol use, and illicit drug use in the last 12 months), sexual behavior in the last 12 months (population sample, type of partnership, number of steady and/or casual partners, gender of partners, sexual practices, condom use, lubricant use, characteristics associated with partnership vulnerability), and health history (type of exposure, first HIV and syphilis test in life, syphilis test results, and STI diagnosis in the last 12 months).

The data were analyzed using descriptive analysis, such as frequency distribution, measures of central tendency (arithmetic mean and median) and position (standard deviation – SD). To assess the association between the variable of interest (young people versus adults) and the other variables, Pearson’s chi- square or Fisher’s exact tests were applied. In cases where tables larger than 2x2 showed a statistically significant association, standardized and adjusted residual analysis was used to verify the pattern of interdependence between the variables. Residuals greater than 1.96 indicated a positive association, while values less than –1.96 showed a negative association. For all analyses, a significance level of 5% was adopted.

### Ethical Aspects

The research was approved by the Municipal Health Secretariat of Ribeirão Preto, under official letter no. 2,444/2021, and subsequently submitted to the Research Ethics Committee of the Ribeirão Preto School of Nursing, University of São Paulo (EERP-USP), and approved according to opinion no. 6,011,234.

## RESULTS

Between 2018 and 2021, 231 people tested positive for HIV at the municipality’s TCCs, 84 of whom were young people and 147 were adults. Among the young people studied, the mean age was 21.6 years (SD = ±2.2 years) and the median age was 22 years, while for adults, the mean age was 32.6 years (SD = ±7.5 years) and the median age was 29 years.

Sociodemographic variables showed that most of the people included in the study were cisgender men (85.3%), white (51.2%), single (82.0%), had completed high school (53.8%), employed (89.3%), used alcohol (72.2%), and did not use illicit drugs (61.3%) in the past 12 months. A statistically significant association was identified between young people who were single, had completed high school, and were students, as well as an association between adults who were married/in a stable relationship, had completed higher education, and were employed (Table 1).

**Table 1:** Distribution of young people and adults with reactive HIV tests (N = 231) according to sociodemographic profile – Ribeirão Preto, SP, Brazil, 2018 to 2021.

Variable		N = 84 Adolescents and young	N = 147 Adults	Total	p-value
		n (%)	n (%)	n (%)	
Gender identity	Cisgender man	74 (88.1)	123 (83.7)	197 (85.3)	0.620**
	Cisgender woman	6 (7.1)	16 (10.9)	22 (9.5)	
	Transgender woman	4 (4.8)	8 (5.4)	12 (5.2)	
Race/color*	White	35 (46.1)	73 (54.1)	108 (51.2)	0.240^†^
	Black	12 (15.8)	12 (8.9)	24 (11.4)	
	Brown	29 (38.2)	47 (34.8)	76 (36.0)	
	Yellow/Indigenous	–	3 (2.2)	3 (1.4)	
Marital status*	Single	69 (93.2)^+^	86 (74.8)^-^	155 (82.0)	**0.002** ^†^
	Married/Civil union	5 (6.8)^-^	22 (19.1)^+^	27 (14.3)	
	Separated/Widowed	–	7 (6.1)^+^	7 (3.7)	
Education*	None	6 (7.4)	16 (11.4)	22 (10.0)	0.004**
	Complete elementary education	17 (21.0)	21 (15.0)	38 (17.2)	
	Complete secondary education	50 (64.2)^+^	67 (47.9)^-^	119 (53.8)	
	Complete higher education	6 (7.4)^-^	36 (25.7)^+^	42 (19.0)	
Employment*	Employed	52 (81.3)^-^	124 (93.2)^+^	176 (89.3)	**0.006** ^†^
	Studying	10 (15.6)^+^	4 (3.0)^-^	14 (7.1)	
	Unemployed	2 (3.1)	5 (3.8)	7 (3.6)	
Alcohol consumption in the last 12 months*	Never used it or used it and no longer uses it	23 (28.4)	36 (27.5)	59 (27.8)	0.885**
	Uses it	58 (71.6)	95 (72.5)	153 (72.2)	
Illegal drug use in the last 12 months*	Never used it or used it and no longer uses it	53 (65.4)	77 (58.8)	130 (61.3)	0.334**
	Uses it	28 (34.6)	54 (41.2)	82 (38.7)	

*The N of adolescents, young, and adults differed in these variables, since blank/ignored/not applicable data were not considered in the analyses;

**Chi-square test;

^†^Fisher's exact test;

^+^standardized and adjusted residuals above 1.96 indicated a positive association;

^-^residuals below –1.96 indicated a negative association.

Source: Prepared by the authors, 2023.

Regarding sexual relations in the last 12 months, the majority (71.2%) were MSM and 48.6% had both a steady partner and occasional partners. Among those with a steady partner, most (73.8%) had a relationship with “one” steady partner, while among those who had occasional partners, most (83.7%) had relationships with “more than one” person in the last 12 months. When all cases were analyzed, it was observed that the majority had relationships only with men (79.7%), engaged in receptive anal sex (75.4%), insertive anal sex (68.8%), receptive oral sex (76.9%), and insertive oral sex (76.1%). Study participants reported that they sometimes used condoms (78.3%) and sometimes used lubricant (56.9%). Regarding sexual partners, it was observed that 27.6% of them were with people living with HIV/STIs, 14.6% had other partners, and 17.6% used illicit drugs. An association was also found between young people and being MSM, as well as between adults, being heterosexual, and having sexual relations with a partner living with HIV/STIs (Table 2).

**Table 2:** Distribution of young people and adults with reactive HIV tests (N = 231) according to sexual behavior in the last 12 months – Ribeirão Preto, SP, Brazil, 2018 to 2021.

Variable		N = 84* Adolescents and young	N = 147* Adults	Total	p-value**
		n (%)	n (%)	n (%)	
Population sample*	Heterosexual	11/78 (14.1)	37/143 (25.9)	48/221 (21.7)	0.043**
	MSM	62/78 (79.5)	96/144 (66.7)	158/222 (71.2)	0.044**
	Sex worker	8/79 (10.1)	6/143 (4.2)	14/222 (6.3)	0.076^†^
Type of partnership	Regular	19 (23.2)	36 (26.5)	55 (25.2)	0.863
	Occasional	22 (26.8)	35 (25.7)	57 (26.1)	
	Regular and occasional	41 (50.0)	65 (47.8)	106 (48.6)	
Number of steady partnerships in the last 12 months	1	30 (66.7)	60 (77.9)	90 (73.8)	0.395
	2	8 (17.8)	9 (11.7)	17 (13.9)	
	3 or 4	7 (15.6)	8 (10.4)	15 (12.3)	
Number of casual partnerships in the last 12 months	1	9 (15.8)	15 (16.5)	24 (16.3)	0.320
	2	5 (8.8)	16 (17.6)	21 (14.3)	
	3–10	23 (40.4)	39 (42.9)	62 (42.2)	
	11–50	13 (22.8)	16 (17.6)	28 (19.0)	
	>50	7 (12.3)	5 (5.5)	12 (8.2)	
Sexual partner in the last 12 months	Men only	69 (84.1)	104 (77.0)	173 (79.7)	0.107
	Women only	5 (6.1)	21 (15.6)	26 (12.0)	
	Men and women	8 (9.8)	10 (7.4)	18 (8.3)	
Sexual activity in the last 12 months	Vaginal	16/79 (20.3)	36/126 (28.6)	52/205 (25.4)	0.183
	Receptive anal	65/69 (82.3)	88/124 (71.0)	153/203 (75.4)	0.068
	Insertive anal	54/76 (71.1)	83/123 (67.5)	137/199 (68.8)	0.597
	Receptive oral	63/77 (81.8)	90/122 (73.8)	153/199 (76.9)	0.190
	Insertive oral	63/76 (82.9)	87/121 (71.9)	150/197 (76.1)	0.078
Condom use in the last 12 months	Always uses condoms	19 (24.4)	24 (20.0)	43 (21.7)	0.467
	Sometimes or never uses condoms	59 (75.6)	96 (80.0)	155 (78.3)	
Lubricant use in the last 12 months	Always uses lubricant	27 (42.2)	35 (43.8)	62 (43.1)	0.851
	Sometimes or never uses lubricant	37 (57.8)	45 (56.2)	82 (56.9)	
Vulnerability of the partner in the last 12 months	Lives with HIV/STI	12/78 (15.4)	43/121 (35.5)	55/199 (27.6)	**0.002**
	Multiple partners	9/78 (11.5)	20/121 (16.5)	29/199 (14.6)	0.330
	Used illicit drugs	13/78 (16.7)	22/121 (18.2)	35/199 (17.6)	0.784

*The N of adolescents, young, and adults differed in all variables, since blank/ignored/not applicable data were not considered in the analyses;

**Chi-square test.

Source: Prepared by the authors, 2023.

The health history of most of the people studied showed unprotected sexual intercourse (81.8%) and having undergone some form of HIV (54.2%) or syphilis (52.7%) testing in their lifetime. Among the results of serological testing among young people and adults, 29.1% were reactive for syphilis and 11.7% had been diagnosed with an STI other than HIV in the past 12 months. There was a statistically significant association between reactive HIV tests and young people who reported having undergone more than one HIV and syphilis test in their lifetime (Table 3).

**Table 3:** Distribution of young people and adults with reactive HIV tests (N = 231) according to health history – Ribeirão Preto, SP, Brazil, 2018 a 2021.

Variable		N = 84* Adolescents and young	N = 147* Adults	Total	p-value**
		n (%)	n (%)	n (%)	
Type of exposure*	Unprotected sex	65/78 (83.3)	115/142 (81.0)	180/220 (81.8)	0.666
	Condom breakage	14/80 (17.5)	22/142 (15.5)	36/222 (16.2)	0.697
	Partner has or has had an STI	12/78 (15.4)	33/142 (23.2)	45/220 (20.5)	0.167
First HIV test*	Yes	4 (25.0)	18 (56.3)	22 (45.8)	**0.041**
	No	12 (75.0)	14 (43.7)	26 (54.2)	
First syphilis test*	Yes	3 (23.1)	16 (59.3)	19 (47.5)	**0.032**
	No	10 (76.9)	11 (40.7)	21 (52.5)	
Syphilis testing*	Reactive	25 (32.9)	34 (26.8)	59 (29.1)	0.352
	Non-reactive	51 (67.1)	90 (73.2)	144 (70.9)	
Diagnosed with an STI in the last 12 months*	Yes	12 (15.0)	13 (9.8)	25 (11.7)	0.251
	No	68 (85.0)	120 (90.2)	188 (88.3)	

*The N of adolescents, young, and adults differed in all variables, since blank/ignored/not applicable data were not considered in the analyses;

**Chi-square test.

Source: Prepared by the authors, 2023.

## DISCUSSION

In the present study, it was found that most of the records analyzed corresponded to individuals under 30 years of age, showing that HIV infection has affected populations in the prime of their youth and productive lives. Younger people tend to have lower levels of education and less knowledge about HIV, in addition to being exposed to different situations of vulnerability. In this context, health actions aimed at adolescents are necessary, with a focus on building knowledge that will continue into adulthood^(14)^.

It should be noted that five individuals between the ages of 15 and 17 tested positive for HIV, demonstrating the importance and relevance of TCCs as strategic services for HIV diagnosis in young populations. In relation to the identified age group, which is still in the educational system, there is an urgent need to implement innovative activities, anchored in the promotion of sexual and reproductive health and in prevention and timely diagnosis strategies through intersectoral approaches to Primary Health Care (PHC) and the inclusion of schools and other social spaces^(15)^.

In addition, health services must ensure that information on STI and HIV prevention is presented in a clear and objective manner, addressing aspects such as prophylactic measures, the immunological window at the time of testing, and possibilities for viral suppression^(16,17)^.

When analyzing the other sociodemographic characteristics, it was noted that the variables related to marital status, education, and employment status reflected the expected profile among the groups studied. Thus, a significant portion of young individuals were single, were studying, and had a lower level of education when compared to adults. It was also observed that many young people were employed, demonstrating that HIV infection did not constitute an impediment to work activities among adults and young people.

The other sociodemographic characteristics, whose associations were not related to the groups studied, deserve mention due to the high number of cisgender men who reported being white among those living with HIV, reinforcing that recognition of these vulnerabilities can lead to strategies that are more targeted to specific social groups, as well as being more assertive in the prevention and diagnosis of new HIV cases^(18)^.

Regarding the use of psychoactive substances, around 70% of the people studied reported drinking alcohol and 40% used illicit drugs, which can be considered high rates. It is understood that the use of these substances influences inconsistent condom use and, consequently, increases vulnerability to STIs and HIV. Thus, it is essential that the population understand the risks involved in sexual practices under the influence of alcohol and drugs, building knowledge that favors safer behaviors^(19)^. The prevention of alcohol and psychoactive substance use must be linked to public health, education, and social protection policies^(19,20)^, ensuring a broader approach to the problem at hand.

In variables related to sexual behavior, being young was associated with men who have sex with men and adults being heterosexual. This finding highlights the high vulnerability of the group of men who have sex with men, which has historically been more affected by HIV in Brazil, although an increase in cases among heterosexuals has also been observed^(3,21)^.

Another characteristic of vulnerability among adults was the presence of partners living with HIV and other STIs. In this case, strategies targeting these individuals may encourage more consistent use of lubricant gel and condoms to reduce the risk of transmission of such infections. The significant number of study participants who reported sometimes or never using these methods highlights the need for health initiatives that promote awareness of the risks of HIV infection during sexual practices, in addition to counseling before and after testing.

With regard to sexual behavior, many individuals studied mentioned having occasional and multiple partners, which increases exposure to HIV. Given this, it is essential to guarantee the rights to safer and healthier sexual practices among young people, making them aware of the risks associated with early onset of sexual practices and relationships established throughout life^(19)^.

In addition, a significant proportion of individuals had sex only with men, demonstrating that this group may be the largest transmitter of HIV, especially when sexual activity occurs via anal intercourse. It is worth noting the high number of oral sex practices, which should be performed with the use of condoms, even though they present a low risk of HIV transmission when compared to other sexual practices. During counseling sessions, it is important to clarify the risks associated with different sexual practices, emphasizing that vulnerability to HIV infection varies according to sexual practice, with higher chances of virus transmission in receptive anal sex, insertive anal sex, receptive vaginal sex, insertive vaginal sex, and oral sex, respectively^(22)^.

In their health history, young people were associated with having already undergone other HIV and syphilis tests in their lives before diagnosis, which indicates a possible spread of testing actions in this population. This contributes to the achievement of the 95–95–95 strategy goals, which aim for 95% of people living with HIV to know their serological status, 95% of people who know their serological status to be on ART, and 95% of people on ART to have suppressed viral loads^(23)^, making them undetectable and untransmittable.

Still regarding health history, most participants reported exposure to HIV due to unprotected sex, which also increases vulnerability to other STIs, such as syphilis, diagnosed in around 30% of the sample. These data reinforce the need for health education activities among this population, enabling the adoption of safer practices as part of the prevention of these infections, which should also include the distribution of condoms, lubricants, and counseling about HIV.

Another effective approach to HIV prevention is the combined prevention mandala, which should be adapted to individual or collective realities and preferences. This strategy includes different actions, such as post-exposure prophylaxis (PEP) and pre-exposure prophylaxis (PrEP), prevention of vertical transmission, immunization against hepatitis B and HPV, harm reduction, diagnosis and treatment of STIs and viral hepatitis, consistent use of condoms with lubricant, treatment of all people living with HIV, and regular testing for HIV, STIs, and viral hepatitis^(24,25)^.

The availability of these technologies reinforces the importance of adapting them to the population and individual profile of each region, taking into account the reality and sexual behavior of people and their partners. In this context, it is also important to expand rapid HIV testing in extramural spaces, such as schools, saunas, parties, squares, and other places where sexual encounters and practices occur, favoring access to prevention and early diagnosis^(26,27)^.

In order to contribute to the reduction of HIV transmission, nursing plays an important role in TCCs, since its activities include training the multidisciplinary team on issues related to virus prevention and health promotion, providing individual and collective care and counseling, and requesting HIV and other STI tests. Thus, nursing should be present throughout the care process to provide guidance and answer questions, from reception to post-consultation^(28)^.

The analysis of the profile and behavior of individuals who test positive for HIV is essential to guide strategies that reduce virus transmission among specific populations and those in situations of greater vulnerability. This understanding allows for the adaptation of prevention measures on an individual and collective basis, with a focus on person-centered care, enabling health promotion actions to be aligned with public policies that address STI and HIV prevention in a cross-cutting manner, in addition to promoting awareness about harm reduction, consistent distribution and use of condoms, application of lubricant gel, and encouragement of serological testing. Beyond the work of TCCs, the importance of coordinating actions through PHC stands out, given the complexity and diversity of factors associated with HIV infection among young people and adults. PHC should be integrated with other levels of care in the RAS, establishing connections with intersectoral policies and actions to strengthen the response to reducing HIV transmission.

Among the limitations of this study, we highlight a possible information bias resulting from the lack of validation of the TCC service form, which may have compromised its adequacy to the participants’ level of knowledge. In addition, a second limitation of the study is the fact that its population is small and restricted to a single municipality, which may prevent the generalization of findings and the identification of vulnerabilities that plague people affected by HIV in Brazil.

## CONCLUSION

In the present study, it was observed that most of the people included were under 30 years of age, were cisgender men, white, employed, used alcohol, used condoms and lubricant inconsistently, had occasional and multiple partners, and had sex exclusively with other men. Among young people, the association with being men who have sex with men and having already undergone other tests for HIV and syphilis throughout their lives stood out. Among adults, it was more common to identify as heterosexual and to have partners living with HIV or other STIs. Understanding the profile and behavior of these HIV-positive individuals is essential to support health strategies aimed at reducing virus transmission among specific populations and those in situations of greater vulnerability.

## DATA AVAILABILITY

The data are available in an electronic repository and can be accessed through the following link: https://repositorio.usp.br/item/003225747.

## References

[1] Joint United Nations Programme on HIV/AIDS. (2023). The path that ends AIDS: UNAIDS global AIDS update 2023
[Internet]..

[2] Joint United Nations Programme on HIV/AIDS. (2023). World AIDS day 2023 [Internet]..

[3] Brasil. (2023). Ministério da Saúde. Secretaria de Vigilância em Saúde. Boletim
epidemiológico - HIV e Aids 2023 [Internet]..

[4] Shuper PA, Joharchi N, Monti PM, Loutfy M, Rehm J (2017). Acute alcohol consumption directly increases HIV transmission
risk: a randomized controlled experiment. J Acquir Immune Defic Syndr..

[5] Pinyaphong J, Srithanaviboonchai K, Chariyalertsak S, Phornphibul P, Tangmunkongvorakul A, Musumari PM (2018). Inconsistent condom use among male university students in
Northern Thailand. Asia Pac J Public Health..

[6] Barbosa KF, Batista AP, Nacife MBPSL, Vianna VN, Oliveira WW, Machado EL (2019). Fatores associados ao não uso de preservativo e prevalência de
HIV, hepatites virais B e C e sífilis: estudo transversal em comunidades
rurais de Ouro Preto, Minas Gerais, entre 2014 e 2016. Epidemiol Serv Saude.

[7] Mann JM, Tarantola D, Netter TW (1992). AIDS in the world..

[8] Ayres JR (2023). Vulnerabilidade, cuidado e integralidade: reconstruções
conceituais e desafios atuais para as políticas e práticas de cuidado em
HIV/Aids. Saúde Debate..

[9] Gomes MT, Oliveira DC, Santos EI, Santo CCE, Valois BRG, Pontes APM (2012). As facetas do convívio com o HIV: formas de relações sociais e
representações sociais da AIDS para pessoas soropositivas
hospitalizadas. Esc Anna Nery..

[10] Harrison A, Colvin CJ, Kuo C, Swartz A, Lurie M (2015). Sustained high HIV incidence in young women in Southern Africa:
social, behavioral, and structural factors and emerging intervention
approaches. Curr HIV/AIDS Rep..

[11] Santelli JS, Mathur S, Song X, Huang T, Wei Y, Lutalo T (2015). Rising school enrollment and declining HIV and pregnancy risk
among adolescents in Rakai District, Uganda, 1994–2013. Glob Soc Welf..

[12] Brasil. (2010). Ministério da Saúde. Secretaria de Atenção à Saúde. Departamento de
Ações Programáticas Estratégicas. Diretrizes Nacionais para a atenção
integral à saúde de adolescentes e jovens na promoção, proteção e
recuperação da saúde – Série A, normas e manuais técnicos
[Internet]..

[13] Joint United Nations Programme on HIV/AIDS. (2021). Young people and HIV [Internet]..

[14] Pavinati G, Lima LV, Paiano M, Jaques AE, Magnabosco GT (2023). Contextos de vulnerabilidade de adolescentes que (con)vivem com
HIV: uma revisão integrativa. Rev Cuid.

[15] Bossonario PA, Ferreira MRL, Andrade RLDP, Sousa KDLD, Bonfim RO, Saita NM (2022). Risk factors for HIV infection among adolescents and the youth: a
systematic review. Rev Lat Am Enfermagem.

[16] Guimarães MDC, Kendall C, Magno L, Rocha GM, Knauth DR, Dourado I (2019). HIV/AIDS knowledge among MSM in Brazil: a challenge for public
policies.. Rev Bras Epidemiol..

[17] Silva LCLD, Ribeiro LCS, Ferreira JDA, Abrantes MSDAP, Dias DEM, Santos MGMDC (2020). Conhecimento de homens jovens sobre infecção pelo HIV e fatores
associados. Rev Baiana Enferm..

[18] Pires DRF, Zuccaro N, Souza FL, Périssé ARS (2023). Projeto de ação integrativa serviço-academia para testagem para
IST/HIV em unidades móveis em Niterói, Rio de Janeiro. Saúde Debate..

[19] Garcia EC, Costa IR, Oliveira RC, Silva CR, Góis AR, Abrão FM (2021). Representações sociais de adolescentes sobre a transmissão do
HIV/AIDS nas relações sexuais: vulnerabilidades e riscos.. Esc Anna Nery..

[20] Pedroso RT, Juhásová MB, Hamann EM (2019). A ciência baseada em evidências nas políticas públicas para
reinvenção da prevenção ao uso de álcool e outras drogas. Interface..

[21] Leite DS (2020). A AIDS no Brasil: mudanças no perfil da epidemia e
perspectivas. Braz J Dev..

[22] Centers for Disease Control and Prevention. (2015). National center for HIV/AIDS, Viral Hepatitis, STD, and
Tuberculosis prevention.. Atlanta: Centers for Disease Control and Prevention.

[23] Joint United Nations Programme on HIV/AIDS. (2020). Seizing the moment: tackling entrenched inequalities to end epidemics
[Internet]..

[24] Brasil. (2022). Ministério da Saúde. Protocolo clínico e diretrizes terapêuticas para
Profilaxia Pré-exposição (Prep) de risco à infecção pelo HIV
[Internet]..

[25] Sousa LRM, Elias HC, Caliari JDS, Oliveira ACD, Gir E, Reis RK (2023). Uso inconsistente do preservativo masculino entre homens HIV
negativos que fazem sexo com homens. Rev Lat Am Enfermagem..

[26] Brasil. (2018). Ministério da Saúde. Manual técnico para o diagnóstico da infecção pelo
HIV em adultos e crianças [Internet]..

[27] Torres RMC, Bastos LS, Gomes MFC, Moreira RI, Périssé ARS, Cruz MM (2021). Avaliação de risco para infecção HIV em homens que fazem sexo com
homens e a contribuição das redes de parceiros sexuais. Cien Saude Colet..

[28] Augusto PS, Santos TR, Oliveira CB, Lima VB, Costa ALC, Costa DM (2024). Health management of an HIV testing and counseling center:
nursing contributions. Rev Bras Enferm..

