# Supplementary material for: Comparative study of the vulnerability profile of young people and adults with reactive HIV tests
Source: Rev Esc Enferm USP. 2025 Oct 13;59:e20250119. doi: 10.1590/1980-220X-REEUSP-2025-0119en (PMC12533483; doi:10.1590/1980-220X-REEUSP-2025-0119en)
Supplement: Translation

# Translation: Estudo comparativo do perfil de vulnerabilidade de jovens e adultos com teste reagente para o HIV*

**DOI:** 10.1590/1980-220X-REEUSP-2025-0119pt

**Authors:** Pedro Augusto Bossonario, Rubia Laine de Paula Andrade, Jaqueline Garcia de Almeida Ballestero, Gabriela Tavares Magnabosco, Gabriel Pavinati, Aline Aparecida Monroe

**Affiliations:** 1 Universidade Estadual de Maringá, Departamento de Enfermagem, Maringá, PR, Brasil.; 2 Universidade de São Paulo, Escola de Enfermagem de Ribeirão Preto, Departamento de Enfermagem Materno-Infantil e Saúde Pública, Ribeirão Preto, SP, Brasil.

**Keywords:** HIV, Perfil de Saúde, Teste de HIV, Comportamento Sexual

## Abstract

**Objetivo:**

Identificar e comparar o perfil de vulnerabilidade de jovens e adultos com teste reagente para o vírus da imunodeficiência humana, em um município brasileiro de grande porte.

**Método:**

Estudo epidemiológico descritivo, com 84 jovens e 147 adultos que procuraram um dos Centros de Testagem e Aconselhamento municipal entre 2018 e 2021 e obtiveram resultado reagente para testagem do vírus da imunodeficiência humana. Os dados foram coletados por um formulário estruturado autoaplicável. Foram realizadas análise descritiva dos dados, teste Qui-quadrado, exato de Fisher e análise de resíduo padronizado e ajustado.

**Resultados:**

Houve associação estatisticamente significativa entre ser jovem e solteiro, ter ensino médio completo, ser estudante, ser homem que faz sexo com homens e não ter realizado teste do vírus da imunodeficiência humana e sífilis até o momento do diagnóstico, além disso, houve associação de adultos com situação conjugal casado/união estável, ensino superior completo, empregados, heterossexuais e contactantes de parceria com infecção sexualmente transmissível.

**Conclusão:**

Conhecer o perfil de vulnerabilidade de pessoas com testagem reagente para o vírus da imunodeficiência humana contribui para orientar ações de saúde pública voltadas ao controle da transmissão do vírus entre grupos populacionais que necessitam de maior atenção.

## INTRODUÇÃO

Aproximadamente 39 milhões de pessoas viviam com o vírus da imunodeficiência humana (HIV) no mundo em 2022, das quais 29,8 milhões estavam em tratamento antirretroviral, 1,3 milhões foram diagnosticadas como casos novos, 37,5 milhões tinham mais de 15 anos de idade e 2,2 milhões residiam na América Latina^(1,2)^. Esse contexto reforça o HIV como um importante problema de saúde pública no mundo, colocando-o como protagonista de políticas para eliminação da infecção.

No Brasil, entre 2007 e 2022, foram notificados 469.357 casos novos de HIV no Sistema de Informação de Agravos de Notificação (SINAN), com registro anual superior a 40 mil casos a partir de 2015, exceto em 2020, período da pandemia da covid-19^(3)^. Em 2022, foram registrados 43.403 novos casos de HIV, enquanto entre 2018 e 2021, período correspondente ao presente estudo, 169.383 pessoas foram notificadas com HIV, das quais 161.060 tinham entre 15 a 59 anos^(3)^.

Entre jovens e adultos, observa-se comportamentos que aumentam a vulnerabilidade à infecção pelo HIV, como uso de drogas lícitas e/ou ilícitas, uso inconsistente de preservativo, parcerias múltiplas, desigualdade de gênero e outras condições que aumentam a vulnerabilidade ao vírus^(4–6)^.

Nesse sentido, conhecer o perfil das pessoas com testagem reagente para o HIV permite a identificação de indivíduos em situação de vulnerabilidade, especialmente no que tange aos aspectos individuais, coletivos e contextuais que, em conjunto, elevam a suscetibilidade a infecções e doenças^(7,8)^. Tal compreensão contribui para o direcionamento de políticas públicas de saúde para a prevenção e controle da transmissão do vírus, além de favorecer o alcance de populações vulnerabilizadas por meio de estratégias adaptadas às realidades das pessoas e dos serviços de saúde.

Historicamente, a compreensão do perfil das pessoas que vivem com HIV evoluiu em três fases distintas da epidemia. A primeira foi marcada pelo predomínio de homens homossexuais com diferentes anos de escolaridade, considerados, à época, como “grupos de risco”. Na segunda fase, passou-se a adotar o conceito de “comportamento de risco”, com ênfase em pessoas que utilizavam drogas injetáveis. A terceira fase, por sua vez, alcançou o conceito de vulnerabilidade, incorporando dimensões individuais, sociais e programáticas, bem como levando em consideração o acometimento de heterossexuais, mulheres, pessoas com baixa escolaridade e a interiorização da aids no território^(9)^.

A despeito de quaisquer classificações adotadas ao longo do tempo, cabe reconhecer que a vulnerabilidade ao HIV possui diversas facetas estruturadas por fatores biológicos, comportamentais, sociais e estruturais, as quais são melhores compreendidas quando observada a distribuição global dos casos de HIV, que se dá, majoritariamente, entre profissionais do sexo, usuários de drogas injetáveis, homens gays e homens que fazem sexo com homens (HSH), mulheres transgênero, clientes de profissionais do sexo e parcerias sexuais de populações-chave. Esses grupos representam a maior parte dos casos, enquanto a população restante corresponde a cerca de 30%^(1,10,11)^.

Por fim, o estado de São Paulo obteve 36.025 casos novos de HIV registrados no SINAN e 32.681 casos de aids incluídos no Sistema de Informação sobre Mortalidade (SIM) durante o período de 2018 e 2022, bem como uma taxa de detecção para aids de 13,7 pessoas a cada 100 mil habitantes no ano de 2022^(3)^. Diante desse cenário, este estudo pretendeu identificar e comparar o perfil de vulnerabilidade de jovens e adultos com teste reagente para o vírus da imunodeficiência humana de um município brasileiro de grande porte.

## MÉTODO

### Tipo de Estudo

Trata-se de um estudo epidemiológico, do tipo descritivo, com dados secundários. Ressalta-se que para elaboração do artigo, as recomendações do *Strengthening the Reporting of Observational Studies in Epidemiology* (STROBE) foram atendidas.

### Local

Considerou-se os cinco Centros de Testagem e Aconselhamento (CTA) de um município paulista de grande porte.

### População e Critérios de Seleção

A população do estudo constitui-se de jovens e adolescentes que procuraram um dos cinco CTA do município estudado para realização de teste rápido ou sorológico para HIV entre os anos de 2018 e 2021. O critério de inclusão estendeu-se a todas as pessoas que tiveram o teste reagente para a infecção. Assim, segundo o Ministério da Saúde do Brasil e outros materiais, a população composta por adolescentes e jovens corresponde à na faixa etária entre 15 e 24 anos, classificação assumida nesta pesquisa^(12,13)^. Para a população adulta, considerou-se as pessoas com idade entre 25 e 59 anos.

### Coleta de Dados

Entre janeiro e junho de 2022, os dados do estudo foram coletados a partir de fonte secundária, constituída pelas Ficha de Atendimento do CTA. O instrumento é autopreenchido pelas pessoas que comparecem ao serviço de saúde e é composto por 42 questões, divididas em oito seções: busca pelo serviço; identificação (dados sociodemográficos); informações para contato; fatores de risco para hepatites; tuberculose (sintomas respiratórios ou contato); uso de substâncias psicoativas; infecções sexualmente transmissíveis (IST); e comportamento sexual.

Outras seis seções da ficha (recorte populacional, tipo de exposição, testagem, conduta, retorno e observações/encerramento) compreendem 10 questões, as quais são preenchidas pelos profissionais de saúde de acordo com as respostas dadas pelos usuários do CTA, para, assim, proceder aos resultados de exames e condutas tomadas após tais resultados. Dessa forma, as respostas do instrumento são preenchidas de forma aberta ou dicotômica e por múltipla escolha.

Para coleta dos dados, considerou-se todas as fichas disponíveis no serviço das pessoas que tiveram testagem reagente durante o ano estipulado, sendo transcritas por meio de um instrumento elaborado pelo software intitulado Research Electronic Data Capture (REDCap).

### Análise e Tratamento dos Dados

No presente estudo, comparou-se o perfil de jovens e adultos segundo variáveis sociodemográficas (identidade de gênero, raça/cor, situação conjugal, escolaridade, trabalho, uso de álcool e/ou de drogas ilícitas nos últimos 12 meses), de comportamento sexual nos últimos 12 meses (recorte populacional, tipo de parceria, número de parcerias fixas e/ou eventuais, gênero das parcerias, prática sexual, uso de preservativo, uso de lubrificante, características associadas à vulnerabilidade da parceria) e de históricos de saúde (tipo de exposição, primeiro teste na vida de HIV e sífilis, resultado da testagem de sífilis e diagnóstico de IST nos último 12 meses).

Os dados foram analisados por meio de análise descritiva, como distribuição de frequência, medidas de tendência central (média aritmética e mediana) e de posição (desvio padrão – dp). Para avaliar a associação entre a variável de interesse (jovens *versus* adultos) e as demais variáveis, aplicou-se os testes qui-quadrado de Pearson ou exato de Fisher. Nos casos em que as tabelas maiores que 2 × 2 apresentavam associação estatisticamente significante, a análise de resíduos padronizados e ajustados fui utilizada para verificar o padrão de interdependência entre as variáveis. Resíduos superiores a 1,96 indicaram associação positiva, enquanto valores inferiores a –1,96 mostraram associação negativa. Para todas as análises, adotou-se um nível de significância de 5%.

### Aspectos Éticos

A pesquisa foi anuída pela Secretaria Municipal de Saúde de Ribeirão Preto, sob o ofício n. 2.444/2021 e, posteriormente, submetida ao Comitê de Ética em Pesquisa da Escola de Enfermagem de Ribeirão Preto, da Universidade de São Paulo (EERP-USP), sendo aprovada segundo parecer n. 6.011.234.

## RESULTADOS

Foram identificadas 231 pessoas com testagem reagente ao HIV entre os anos de 2018 e 2021 nos CTA do município, sendo que 84 eram jovens e 147 eram adultos. Entre os jovens estudados, a idade média foi 21,6 anos (dp = ±2,2 anos) e a mediana de 22 anos, enquanto para os adultos, a média de idade foi de 32,6 anos (dp = ±7,5 anos) e a mediana de 29 anos.

As variáveis sociodemográficas demonstraram que a maioria das pessoas incluídas no estudo eram homens cisgênero (85,3%), de cor branca (51,2%), solteiras (82,0%), com ensino médio completo (53,8%), empregadas (89,3%), utilizou álcool (72,2%) e não usou drogas ilícitas (61,3%) nos últimos 12 meses. Foi identificada associação estatisticamente significativa entre jovens com a situação conjugal solteira, ensino médio completo e estudantes, bem como associação de adultos com situação conjugal casado/união estável, ensino superior completo e estar empregado (Tabela 1).

**Tabela 1:** Distribuição de jovens e adultos com teste reagente para HIV (N = 231) segundo perfil sociodemográfico – Ribeirão Preto, SP, Brasil, 2018 a 2021.

Variável		N = 84 Adolescentes e jovens	N = 147 Adultos	Total	Valor p
		n (%)	n (%)	n (%)	
Identidade de gênero	Homem cisgênero	74 (88,1)	123 (83,7)	197 (85,3)	0,620**
	Mulher cisgênero	6 (7,1)	16 (10,9)	22 (9,5)	
	Mulher transgênero	4 (4,8)	8 (5,4)	12 (5,2)	
Raça/cor*	Branca	35 (46,1)	73 (54,1)	108 (51,2)	0,240^†^
	Preta	12 (15,8)	12 (8,9)	24 (11,4)	
	Parda	29 (38,2)	47 (34,8)	76 (36,0)	
	Amarela/Indígena	–	3 (2,2)	3 (1,4)	
Situação conjugal*	Solteiro	69 (93,2)^+^	86 (74,8)^-^	155 (82,0)	**0,002** ^†^
	Casado/União estável	5 (6,8)^-^	22 (19,1)^+^	27 (14,3)	
	Separado/Viúvo	–	7 (6,1)^+^	7 (3,7)	
Escolaridade*	Nenhuma	6 (7,4)	16 (11,4)	22 (10,0)	0,004**
	Fundamental completa	17 (21,0)	21 (15,0)	38 (17,2)	
	Médio completo	50 (64,2)^+^	67 (47,9)^-^	119 (53,8)	
	Superior completo	6 (7,4)^-^	36 (25,7)^+^	42 (19,0)	
Trabalho*	Empregado	52 (81,3)^-^	124 (93,2)^+^	176 (89,3)	**0,006** ^†^
	Estuda	10 (15,6)^+^	4 (3,0)^-^	14 (7,1)	
	Desempregado	2 (3,1)	5 (3,8)	7 (3,6)	
Álcool nos últimos 12 meses*	Nunca usou ou já usou e não usa mais	23 (28,4)	36 (27,5)	59 (27,8)	0,885**
	Usa	58 (71,6)	95 (72,5)	153 (72,2)	
Uso de drogas ilícitas nos últimos 12 meses*	Nunca usou ou já usou e não usa mais	53 (65,4)	77 (58,8)	130 (61,3)	0,334**
	Usa	28 (34,6)	54 (41,2)	82 (38,7)	

*O N de adolescentes, jovens e adultos diferiu nestas variáveis, uma vez que os dados em brancos/ignorados/não se aplicam não foram considerados nas análises;

**Teste Qui-quadrado;

^†^Teste exato de Fisher;

^+^resíduos padronizados e ajustados acima de 1,96 indicaram associação positiva;

^-^resíduos abaixo de -1,96 indicaram associação negativa.

Fonte: Elaborado pelos autores, 2023.

Quanto às relações sexuais nos últimos 12 meses, a maioria (71,2%) era HSH e 48,6% possuíam parceria fixa e eventual concomitantemente. Entre os casos com parceria fixa, a maioria (73,8%) teve relacionamento com “uma” parceria fixa enquanto os casos que tinham parceria eventual, a maioria (83,7%) se relacionou com “mais de uma” pessoa nos últimos 12 meses. Quando analisado todos os casos, observou-se que a maioria mantinha relacionamento somente com homens (79,7%), faziam sexo anal receptivo (75,4%), anal insertivo (68,8%), oral receptivo (76,9%) e oral insertivo (76,1%). Os participantes do estudo relataram às vezes utilizarem preservativo (78,3%) e às vezes usarem gel lubrificante (56,9%). Em relação às parcerias sexuais, observou- se que 27,6% delas eram com pessoas que viviam com HIV/IST, 14,6% mantinham outras parcerias e 17,6% utilizavam drogas ilícitas. Revelou-se, ainda, associação entre jovens e ser HSH, bem como entre adultos, ser heterossexual e ter relação sexual com parceria que vivia com HIV/IST (Tabela 2).

**Tabela 2:** Distribuição de jovens e adultos com teste reagente para HIV (N = 231) segundo o comportamento sexual últimos 12 meses – Ribeirão Preto, SP, Brasil, 2018 a 2021.

Variável		N = 84* Adolescentes e jovens	N = 147** Adultos	Total	Valor p**
		n (%)	n (%)	n (%)	
Recorte populacional*	Heterossexual	11/78 (14,1)	37/143 (25,9)	48/221 (21,7)	0,043**
	HSH	62/78 (79,5)	96/144 (66,7)	158/222 (71,2)	0,044**
	Profissional do sexo	8/79 (10,1)	6/143 (4,2)	14/222 (6,3)	0,076^†^
Tipo de parceria	Fixa	19 (23,2)	36 (26,5)	55 (25,2)	0,863
	Eventual	22 (26,8)	35 (25,7)	57 (26,1)	
	Fixa e eventual	41 (50,0)	65 (47,8)	106 (48,6)	
Número de parcerias fixas nos últimos 12 meses	1	30 (66,7)	60 (77,9)	90 (73,8)	0,395
	2	8 (17,8)	9 (11,7)	17 (13,9)	
	3 ou 4	7 (15,6)	8 (10,4)	15 (12,3)	
Número de parcerias eventuais nos últimos 12 meses	1	9 (15,8)	15 (16,5)	24 (16,3)	0,320
	2	5 (8,8)	16 (17,6)	21 (14,3)	
	3–10	23 (40,4)	39 (42,9)	62 (42,2)	
	11–50	13 (22,8)	16 (17,6)	28 (19,0)	
	>50	7 (12,3)	5 (5,5)	12 (8,2)	
Parceria sexual nos últimos 12 meses	Somente homens	69 (84,1)	104 (77,0)	173 (79,7)	0,107
	Somente mulheres	5 (6,1)	21 (15,6)	26 (12,0)	
	Homens e mulheres	8 (9,8)	10 (7,4)	18 (8,3)	
Prática sexual nos últimos 12 meses	Vaginal	16/79 (20,3)	36/126 (28,6)	52/205 (25,4)	0,183
	Anal receptivo	65/69 (82,3)	88/124 (71,0)	153/203 (75,4)	0,068
	Anal insertivo	54/76 (71,1)	83/123 (67,5)	137/199 (68,8)	0,597
	Oral receptivo	63/77 (81,8)	90/122 (73,8)	153/199 (76,9)	0,190
	Oral insertivo	63/76 (82,9)	87/121 (71,9)	150/197 (76,1)	0,078
Uso de preservativo nos últimos 12 meses	Sempre utiliza preservativo	19 (24,4)	24 (20,0)	43 (21,7)	0,467
	Às vezes ou nunca utiliza preservativo	59 (75,6)	96 (80,0)	155 (78,3)	
Uso de lubrificante nos últimos 12 meses	Sempre utiliza lubrificante	27 (42,2)	35 (43,8)	62 (43,1)	0,851
	Às vezes ou nunca utiliza lubrificante	37 (57,8)	45 (56,2)	82 (56,9)	
Vulnerabilidade da parceria nos últimos 12 meses	Vive com HIV/IST	12/78 (15,4)	43/121 (35,5)	55/199 (27,6)	**0,002**
	Múltiplas parcerias	9/78 (11,5)	20/121 (16,5)	29/199 (14,6)	0,330
	Usava drogas ilícitas	13/78 (16,7)	22/121 (18,2)	35/199 (17,6)	0,784

*O N de adolescentes, jovens e adultos diferiu em todas as variáveis, uma vez que os dados em brancos/ignorados/não se aplicam não foram considerados nas análises;

**Teste qui-quadrado.

Fonte: Elaborado pelos autores, 2023.

O histórico de saúde da maioria das pessoas estudadas mostrou relações sexuais desprotegidas (81,8%) e já ter realizado algum teste de HIV (54,2%) ou de sífilis (52,7%) na vida. Entre os resultados das testagens sorológicas entre jovens e adultos, 29,1% foram reagentes para sífilis e 11,7% tiveram diagnóstico de IST nos últimos 12 meses que não fosse o HIV. Existiu associação estatisticamente significativa entre os testes reagentes para HIV e jovens que relataram já ter realizado mais de um testagem de HIV e sífilis na vida (Tabela 3).

**Tabela 3:** Distribuição de jovens e adultos com teste reagente para HIV (N = 231) segundo históricos de saúde – Ribeirão Preto, SP, Brasil, 2018 a 2021.

Variável		N = 84* Adolescentes e jovens	N = 147* Adultos	Total	Valor p**
		n (%)	n (%)	n (%)	
Tipo de exposição*	Relação sexual desprotegida	65/78 (83,3)	115/142 (81,0)	180/220 (81,8)	0,666
	Rompimento de preservativo	14/80 (17,5)	22/142 (15,5)	36/222 (16,2)	0,697
	Parceria tem ou teve IST	12/78 (15,4)	33/142 (23,2)	45/220 (20,5)	0,167
Primeiro teste na vida de HIV*	Sim	4 (25,0)	18 (56,3)	22 (45,8)	**0,041**
	Não	12 (75,0)	14 (43,7)	26 (54,2)	
Primeiro teste na vida de sífilis*	Sim	3 (23,1)	16 (59,3)	19 (47,5)	**0,032**
	Não	10 (76,9)	11 (40,7)	21 (52,5)	
Testagem de sífilis*	Reagente	25 (32,9)	34 (26,8)	59 (29,1)	0,352
	Não reagente	51 (67,1)	90 (73,2)	144 (70,9)	
Teve diagnóstico de IST nos últimos 12 meses*	Sim	12 (15,0)	13 (9,8)	25 (11,7)	0,251
	Não	68 (85,0)	120 (90,2)	188 (88,3)	

*O N de adolescentes, jovens e adultos diferiu em todas as variáveis, uma vez que os dados em brancos/ignorados/não se aplicam não foram considerados nas análises;

**Teste qui-quadrado.

Fonte: Elaborado pelos autores, 2023.

## DISCUSSÃO

No presente estudo, identificou-se que a maioria dos registros analisados correspondia a indivíduos com menos de 30 anos, evidenciando que a infecção pelo HIV tem acometido populações no auge da juventude e da vida produtiva. As pessoas mais jovens tendem a apresentar menor escolaridade e baixo conhecimento acerca do HIV, além de estarem expostas a diferentes situações de vulnerabilidades. Nesse contexto, tornam-se necessárias ações em saúde voltadas aos adolescentes, com foco na construção de saberes que se perpetuem até a fase adulta^(14)^.

Ressalta-se que foram observados cinco indivíduos com teste reagente ao HIV entre 15 e 17 anos, demonstrando a sexarca precoce e a relevância dos CTA como serviços estratégicos para o diagnostico ao HIV em populações jovens. Em relação à faixa etária identificada, que ainda está inserida no sistema educacional, urge a necessidade de implementação de atividades inovadoras, ancoradas na promoção da saúde sexual e reprodutiva e em estratégias de prevenção e diagnóstico oportuno por meio de abordagens intersetoriais da Atenção Primária à Saúde (APS) e a inclusão de escolas e outros espaços sociais^(15)^.

Ademais, os serviços de saúde devem garantir que as informações sobre prevenção das IST e do HIV sejam construídas de forma clara e objetiva, abordando aspectos como medidas profiláticas, janela imunológica no momento da testagem e possibilidades de supressão viral^(16,17)^.

Ao analisar as demais características sociodemográficas, notou-se que as variáveis relacionadas à situação conjugal, escolaridade e situação laboral refletiram o perfil esperado entre os grupos estudados. Assim, parte significativa dos indivíduos jovens eram solteiros, estavam estudando e apresentaram nível de escolaridade inferior quando comparados aos adultos. Observou-se, ainda, que muitos jovens estavam empregados, demostrando que a infecção pelo HIV não constituiu um impeditivo para a realização das atividades laborais entre adultos e jovens.

As demais características sociodemográficas, cujas associações não tiveram relação com os grupos estudados, merecem destaque devido ao alto número de homens cisgênero e que referiram ser da cor branca entre aqueles vivem com HIV, reforçando que o reconhecimento dessas vulnerabilidades pode levar a estratégias mais direcionadas à determinados grupos sociais, bem como serem mais assertivas na prevenção e diagnóstico de casos novos de HIV^(18)^.

Em relação ao consumo de substâncias psicoativas, cerca de 70% das pessoas estudadas relataram ingerir álcool e 40% fazer uso de drogas ilícitas, valores que podem ser considerados altos. Entende-se que a utilização dessas substâncias influencia no uso inconsistente do preservativo e, por conseguinte, aumenta a vulnerabilidade às IST e ao HIV. Assim, é essencial que a população compreenda os riscos envolvidos nas práticas sexuais sob influência de álcool e drogas, construindo conhecimentos que favoreçam comportamentos mais seguros^(19)^. A prevenção do consumo de álcool e substâncias psicoativas deve estar articulada às políticas públicas de saúde, educação e proteção social^(19,20)^, garantindo uma abordagem ampliada do problema em questão.

Nas variáveis relacionadas ao comportamento sexual, ser jovem foi associado a homens que fazem sexo com outros homens e os adultos a serem heterossexuais. Esse achado evidencia a elevada vulnerabilidade do grupo de homens que fazem sexo com homens, o qual é historicamente mais acometido pelo HIV no Brasil, embora se observe também aumento de casos entre heterossexuais^(3,21)^.

Outra característica de vulnerabilidade entre adultos foi a presença de parcerias que viviam com HIV e outras IST. Nesse caso, estratégias direcionadas a esses indivíduos podem incentivar a utilização mais consistente de gel lubrificante e preservativos para reduzir o risco de transmissão de tais infecções. O número significativo de participantes do estudo que relatou às vezes ou nunca utilizar esses métodos evidencia a necessidade de iniciativas de ações em saúde que promovam o conhecimento acerca dos riscos da infecção ao HIV durante as práticas sexuais, além do aconselhamento em momentos de pré e pós-testagem.

No que se refere ao comportamento sexual, muitos indivíduos estudados mencionaram manter parcerias eventuais e múltiplas, o que aumenta a exposição ao HIV. Diante disso, torna-se essencial a garantia de direitos às práticas sexuais mais seguras e saudáveis entre jovens, conscientizando-os sobre os riscos associados ao início precoce das práticas sexuais e às relações estabelecidas ao longo da vida^(19)^.

Além disso, parte significativa dos indivíduos mantinha relações sexuais somente com homens, demostrando que este grupo pode ser o maior transmissor de HIV, principalmente quando a prática sexual acontece por via anal. Cabe ressaltar o elevado número da prática do sexo oral, a qual deve ser realizada com uso de preservativo, mesmo que apresente baixo risco de transmissão ao HIV quando comparada às demais vias sexuais. Durante as consultas de aconselhamento, é importante esclarecer os riscos associados às diferentes práticas sexuais, destacando que a vulnerabilidade à infecção pelo HIV varia conforme a prática sexual, com maiores chances de transmissão do vírus em sexo anal receptivo, sexo anal insertivo, sexo vaginal receptivo, sexo vaginal insertivo e sexo oral, respectivamente^(22)^.

No histórico de saúde, os jovens estiveram associados a já terem realizado outros testes de HIV e sífilis na vida antes do diagnóstico, o que indica uma possível disseminação das ações de testagem nessa população. Isso contribui para o alcance das metas da estratégia 95–95–95, a qual pretende que 95% das pessoas com HIV saibam seu estado sorológico, 95% das pessoas que sabem seu estado sorológico estejam em TARV e que 95% das pessoas que estejam em TARV tenham carga viral suprimida^(23)^, tornando-os indetectáveis e intransmissíveis.

Ainda quanto ao histórico de saúde, a maior parte dos participantes relataram exposição ao HIV devido à relação sexual desprotegida, o que também aumenta a vulnerabilidade às demais IST, como a sífilis, diagnosticada em cerca de 30% da amostra. Esses dados reforçam a necessidade de atividades de educação em saúde junto à população, possibilitando a adoção de práticas mais seguras como parte da prevenção a essas infecções, a qual também deve incluir a dispensação de preservativos, gel lubrificantes e aconselhamento acerca do HIV.

Outra abordagem eficaz na prevenção do HIV é a mandala da prevenção combinada, que deve ser adaptada às realidades e preferências individuais ou coletivas. Essa estratégia inclui diferentes ações, como profilaxia pós-exposição (PEP) e pré-exposição (PrEP), prevenção da transmissão vertical, imunização contra hepatite B e HPV, redução de danos, diagnóstico e tratamento de IST e hepatites virais, uso consistente de preservativos com gel lubrificante, tratamento de todas as pessoas que vivem com HIV e testagem regular para HIV, IST e hepatites virais^(24,25)^.

A disponibilização dessas tecnologias reforça a importância de adequá-las ao perfil populacional e individual de cada região, levando em conta a realidade e o comportamento sexual das pessoas e suas parcerias. Nesse contexto, destaca-se, ainda, a relevância da ampliação da testagem rápida para HIV em espaços extramuros, como escolas, saunas, festas, praças e outros locais onde ocorrem encontros e práticas sexuais, favorecendo o acesso à prevenção e ao diagnóstico precoce^(26,27)^.

A fim de contribuir com a diminuição da transmissão do HIV, a enfermagem desempenha importante papel nos CTA, uma vez que sua atuação abrange capacitações da equipe multidisciplinar em relação às questões de prevenção do vírus e à promoção da saúde, a realização de atendimentos e aconselhamentos individuais e coletivos, além da solicitação de exames de HIV e outras IST. Dessa forma, a enfermagem deve estar presente em todo processo de cuidado para orientações e esclarecimento de dúvidas, desde o acolhimento até a pós-consulta^(28)^.

A análise do perfil e do comportamento de indivíduos com teste reagente ao HIV é essencial para direcionar estratégias que reduzam a transmissão do vírus entre populações específicas e em maior situação de vulnerabilidade. Essa compreensão permite adaptar as medidas de prevenção de forma individualizada e coletiva, com foco no cuidado centrado na pessoa, possibilitando que as ações de promoção da saúde estejam alinhadas às políticas públicas, que abordam de maneira transversal a prevenção das IST e do HIV, além de promover a conscientização sobre redução de danos, distribuição e uso consistente de preservativos, aplicação de gel lubrificante e incentivo à testagem sorológica. Para além da atuação dos CTA, destaca-se a importância da coordenação de ações pela APS, dada a complexidade e a diversidade dos fatores associados à infecção pelo HIV entre jovens e adultos. A APS deve integrar-se aos demais níveis de atenção da RAS, estabelecendo conexões com políticas e ações intersetoriais para fortalecer a resposta à diminuição na transmissão do HIV.

Entre as limitações deste estudo, ressalta-se um possível viés de informação decorrente da ausência de validação da ficha de atendimento do CTA, o que pode ter comprometido sua adequação ao nível de conhecimento dos participantes. Além disso, pontua-se como uma segunda limitação o fato de sua população ser pequena e restrita a um único município, o que pode impedir a generalização dos achados e a identificação das vulnerabilidades que assolam as pessoas acometidas pelo HIV no Brasil.

## CONCLUSÃO

No presente estudo, observou-se que a maioria das pessoas incluídas tinha menos de 30 anos, era composta por homens cisgênero, de cor branca, com trabalho, que faziam uso de álcool, utilizavam preservativos e gel lubrificante de forma inconsistente, possuíam parcerias eventuais e múltiplas, e se relacionavam exclusivamente com outros homens. Entre os jovens, destacou-se a associação com o fato de serem homens que fazem sexo com homens e já terem realizado outros testes para HIV e sífilis ao longo da vida. Já entre os adultos, foi mais frequente a identificação como heterossexuais e a presença de parcerias vivendo com HIV ou outras IST. Compreender o perfil e o comportamento dessas pessoas com testagem reagente para o HIV é fundamental para subsidiar estratégias de saúde direcionadas à redução da transmissão do vírus entre populações específicas e em maior situação de vulnerabilidade.

## DISPONIBILIDADE DE DADOS

Os dados encontram-se disponibilizados em repositório eletrônico, acessível por meio do seguinte link: https://repositorio.usp.br/item/003225747.

# Translation: Estudio comparativo del perfil de vulnerabilidad de jóvenes y adultos con prueba reactiva al VIH*

**DOI:** 10.1590/1980-220X-REEUSP-2025-0119es

**Authors:** Pedro Augusto Bossonario, Rubia Laine de Paula Andrade, Jaqueline Garcia de Almeida Ballestero, Gabriela Tavares Magnabosco, Gabriel Pavinati, Aline Aparecida Monroe

**Affiliations:** 1 Universidade Estadual de Maringá, Departamento de Enfermagem, Maringá, PR, Brasil.; 2 Universidade de São Paulo, Escola de Enfermagem de Ribeirão Preto, Departamento de Enfermagem Materno-Infantil e Saúde Pública, Ribeirão Preto, SP, Brasil.

**Keywords:** VIH, Perfil de Salud, Prueba del VIH, Conducta Sexual

## Abstract

**Objetivo:**

Identificar y comparar el perfil de vulnerabilidad de jóvenes y adultos con prueba reactiva al virus de la inmunodeficiencia humana en un municipio brasileño de gran tamaño.

**Método:**

Estudio epidemiológico descriptivo con 84 jóvenes y 147 adultos que acudieron a uno de los Centros Municipales de Pruebas y Asesoramiento entre 2018 y 2021 y obtuvieron un resultado reactivo en la prueba del virus de la inmunodeficiencia humana. Los datos se recopilaron mediante un formulario estructurado autoaplicable. Se realizaron análisis descriptivos de los datos, pruebas Chi-cuadrado, exactas de Fisher y análisis de residuos estandarizados y ajustados.

**Resultados:**

Se observó una asociación estadísticamente significativa entre ser joven y soltero, tener estudios secundarios completos, ser estudiante, ser hombre que tiene relaciones sexuales con hombres y no haberse realizado la prueba del virus de la inmunodeficiencia humana ni de sífilis hasta el momento del diagnóstico. Además, se observó una asociación entre adultos con estado civil casado/unión estable, estudios superiores completos, empleados, heterosexuales y personas en contacto con parejas con infecciones de transmisión sexual.

**Conclusión:**

Conocer el perfil de vulnerabilidad de las personas con resultados positivos en las pruebas del virus de la inmunodeficiencia humana contribuye a orientar las acciones de salud pública destinadas a controlar la transmisión del virus entre los grupos de población que requieren mayor atención.

## INTRODUCCIÓN

Aproximadamente 39 millones de personas vivían con el virus de la inmunodeficiencia humana (VIH) en el mundo en 2022, de las cuales 29,8 millones estaban en tratamiento antirretroviral, 1,3 millones fueron diagnosticadas como casos nuevos, 37,5 millones tenían más de 15 años y 2,2 millones residían en América Latina^(1,2)^. Este contexto refuerza el VIH como un importante problema de salud pública en el mundo, situándolo como protagonista de las políticas para la eliminación de la infección.

En Brasil, entre 2007 y 2022, se notificaron 469 357 nuevos casos de VIH en el Sistema de Información de Notificación de Enfermedades (SINAN), con un registro anual superior a 40 000 casos a partir de 2015, excepto en 2020, periodo de la pandemia de COVID-19^(3)^. En 2022, se registraron 43 403 nuevos casos de VIH, mientras que entre 2018 y 2021, periodo correspondiente al presente estudio, se notificaron 169 383 personas con VIH, de las cuales 161 060 tenían entre 15 y 59 años^(3)^.

Entre los jóvenes y adultos se observan comportamientos que aumentan la vulnerabilidad a la infección por el VIH, como el consumo de drogas lícitas y/o ilícitas, el uso inconsistente de preservativos, las relaciones sexuales con múltiples parejas, la desigualdad de género y otras condiciones que aumentan la vulnerabilidad al virus^(4–6)^.

En este sentido, conocer el perfil de las personas con resultados positivos en las pruebas del VIH permite identificar a los individuos en situación de vulnerabilidad, especialmente en lo que se refiere a los aspectos individuales, colectivos y contextuales que, en conjunto, aumentan la susceptibilidad a las infecciones y enfermedades^(7,8)^. Esta comprensión contribuye a orientar las políticas públicas de salud para la prevención y el control de la transmisión del virus, además de favorecer el alcance de las poblaciones vulnerables mediante estrategias adaptadas a las realidades de las personas y los servicios de salud.

Históricamente, la comprensión del perfil de las personas que viven con el VIH ha evolucionado en tres fases distintas de la epidemia. La primera se caracterizó por el predominio de hombres homosexuales con diferentes niveles de escolaridad, considerados en ese momento como «grupos de riesgo». En la segunda fase, se adoptó el concepto de «comportamiento de riesgo», con énfasis en las personas que consumían drogas inyectables. La tercera fase, por su parte, alcanzó el concepto de vulnerabilidad, incorporando dimensiones individuales, sociales y programáticas, así como teniendo en cuenta el impacto en los heterosexuales, las mujeres, las personas con bajo nivel educativo y la interiorización del VIH/SIDA en el territorio^(9)^.

Independientemente de las clasificaciones adoptadas a lo largo del tiempo, hay que reconocer que la vulnerabilidad al VIH tiene diversas facetas estructuradas por factores biológicos, conductuales, sociales y estructurales, que se comprenden mejor cuando se observa la distribución global de los casos de VIH, que se da, en su mayoría, entre profesionales del sexo, usuarios de drogas inyectables, hombres gays y hombres que tienen sexo con hombres (HSH), mujeres transgénero, clientes de profesionales del sexo y parejas sexuales de poblaciones clave. Estos grupos representan la mayor parte de los casos, mientras que la población restante corresponde a alrededor del 30%^(1,10,11)^.

Por último, el estado de São Paulo registró 36 025 nuevos casos de VIH en el SINAN y 32 681 casos de sida incluidos en el Sistema de Información sobre Mortalidad (SIM) durante el periodo comprendido entre 2018 y 2022, así como una tasa de detección del sida de 13,7 personas por cada 100 000 habitantes en el año 2022^(3)^. Ante este panorama, el presente estudio tuvo como objetivo identificar y comparar el perfil de vulnerabilidad de jóvenes y adultos con prueba reactiva al virus de la inmunodeficiencia humana en un municipio brasileño de gran tamaño.

## MÉTODO

### Tipo de Estudio

Se trata de un estudio epidemiológico, de tipo descriptivo, con datos secundarios. Cabe destacar que para la elaboración del artículo se han seguido las recomendaciones del *Strengthening the Reporting of OBservational studies in Epidemiology* (STROBE).

### Lugar

Se consideraron los cinco Centros de Test y Asesoramiento (CTA) de un municipio grande de São Paulo.

### Población y Criterios de Selección

La población del estudio está formada por jóvenes y adolescentes que acudieron a uno de los cinco CTA del municipio estudiado para realizarse una prueba rápida o serológica del VIH entre los años 2018 y 2021. El criterio de inclusión se extendió a todas las personas que dieron positivo en la prueba de infección. Así, según el Ministerio de Salud de Brasil y otros materiales, la población compuesta por adolescentes y jóvenes corresponde al grupo de edad comprendido entre los 15 y los 24 años, clasificación adoptada en esta investigación^(12,13)^. Para la población adulta, se consideraron las personas de entre 25 y 59 años.

### Recolección de Datos

Entre enero y junio de 2022, los datos del estudio se recolectaron a partir de una fuente secundaria, constituida por la Ficha de Atención del CTA.

El instrumento es autoadministrado por las personas que acuden al servicio de salud y consta de 42 preguntas, divididas en ocho secciones: búsqueda del servicio; identificación (datos sociodemográficos); información de contacto; factores de riesgo de hepatitis; tuberculosis (síntomas respiratorios o contacto); consumo de sustancias psicoactivas; infecciones de transmisión sexual (ITS); y comportamiento sexual.

Otras seis secciones de la ficha (segmentación de la población, tipo de exposición, pruebas, conducta, retorno y observaciones/cierre) comprenden 10 preguntas, que son rellenadas por los profesionales de la salud de acuerdo con las respuestas dadas por los usuarios del CTA, para así proceder a los resultados de los exámenes y las conductas tomadas tras dichos resultados. De esta forma, las respuestas del instrumento se completan de forma abierta o dicotómica y mediante selección múltiple.

Para la recolección de datos, se consideraron todas las fichas disponibles en el servicio de las personas que tuvieron resultados reactivos durante el año estipulado, y se transcribieron mediante un instrumento elaborado por el software denominado Research Electronic Data Capture (REDCap).

### Análisis y Tratamiento de los Datos

En el presente estudio, se comparó el perfil de jóvenes y adultos según variables sociodemográficas (identidad de género, raza/color, situación conyugal, escolaridad, trabajo, consumo de alcohol y/o drogas ilícitas en los últimos 12 meses), comportamiento sexual en los últimos 12 meses (muestra poblacional, tipo de pareja, número de parejas estables y/o ocasionales, género de las parejas, prácticas sexuales, uso de preservativos, uso de lubricantes, características asociadas a la vulnerabilidad de la pareja) y de antecedentes de salud (tipo de exposición, primera prueba de VIH y sífilis en la vida, resultado de la prueba de sífilis y diagnóstico de ITS en los últimos 12 meses).

Los datos se analizaron mediante análisis descriptivo, como distribución de frecuencias, medidas de tendencia central (media aritmética y mediana) y de posición (desviación estándar – DE). Para evaluar la asociación entre la variable de interés (jóvenes frente a adultos) y las demás variables, se aplicaron las pruebas Chi-cuadrado de Pearson o exacta de Fisher. En los casos en que las tablas mayores que 2 × 2 presentaban una asociación estadísticamente significativa, se utilizó el análisis de residuos estandarizados y ajustados para verificar el patrón de interdependencia entre las variables. Los residuos superiores a 1,96 indicaron una asociación positiva, mientras que los valores inferiores a –1,96 mostraron una asociación negativa. Para todos los análisis, se adoptó un nivel de significación del 5%.

### Aspectos Éticos

La investigación fue autorizada por la Secretaría Municipal de Salud de Ribeirão Preto, mediante el oficio n.º 2.444/2021, y posteriormente sometida al Comité de Ética en Investigación de la Escuela de Enfermería de Ribeirão Preto, de la Universidad de São Paulo (EERP-USP), siendo aprobada según el dictamen n.º 6.011.234.

## RESULTADOS

Se identificaron 231 personas con pruebas reactivas al VIH entre los años 2018 y 2021 en los CTA del municipio, de las cuales 84 eran jóvenes y 147 eran adultos. Entre los jóvenes estudiados, la edad media fue de 21,6 años (DE = ±2,2 años) y la mediana de 22 años, mientras que para los adultos, la edad media fue de 32,6 años (DE = ±7,5 años) y la mediana de 29 años.

Las variables sociodemográficas mostraron que la mayoría de las personas incluidas en el estudio eran hombres cisgénero (85,3%), de raza blanca (51,2%), solteros (82,0%), con estudios secundarios completos (53,8%), empleadas (89,3%), consumían alcohol (72,2%) y no consumían drogas ilícitas (61,3%) en los últimos 12 meses. Se identificó una asociación estadísticamente significativa entre los jóvenes solteros, con estudios secundarios completos y estudiantes, así como una asociación entre los adultos casados/en unión libre, con estudios superiores completos y empleados (Tabla 1).

**Tabla 1:** Distribución de jóvenes y adultos con prueba reactiva al VIH (N = 231) según perfil sociodemográfico – Ribeirão Preto, SP, Brasil, 2018 a 2021.

Variable		N = 84 Adolescentes y jóvenes	N = 147 Adultos	Total	Valor p
		n (%)	n (%)	n (%)	
Identidad de género	Hombre cisgénero	74 (88,1)	123 (83,7)	197 (85,3)	0,620**
	Mujer cisgénero	6 (7,1)	16 (10,9)	22 (9,5)	
	Mujer transgénero	4 (4,8)	8 (5,4)	12 (5,2)	
Raza/color*	Blanca	35 (46,1)	73 (54,1)	108 (51,2)	0,240†
	Negra	12 (15,8)	12 (8,9)	24 (11,4)	
	Parda	29 (38,2)	47 (34,8)	76 (36,0)	
	Amarilla/Indígena	–	3 (2,2)	3 (1,4)	
Situación conyugal*	Soltero	69 (93,2)+	86 (74,8)^-^	155 (82,0)	**0,002** ^†^
	Casado/Unión estable	5 (6,8)^-^	22 (19,1)^+^	27 (14,3)	
	Separado/Viudo	–	7 (6,1)+	7 (3,7)	
Escolaridad*	Ninguna	6 (7,4)	16 (11,4)	22 (10,0)	0,004**
	Fundamental completa	17 (21,0)	21 (15,0)	38 (17,2)	
	Media completa	50 (64,2)^+^	67 (47,9)^-^	119 (53,8)	
	Superior completa	6 (7,4)^-^	36 (25,7)^+^	42 (19,0)	
Trabajo*	Empleado	52 (81,3)^-^	124 (93,2)^+^	176 (89,3)	**0,006** ^†^
	Estudia	10 (15,6)^+^	4 (3,0)^-^	14 (7,1)	
	Desempleado	2 (3,1)	5 (3,8)	7 (3,6)	
Alcohol nos últimos 12 meses*	Nunca usó o ya usó y no usa más	23 (28,4)	36 (27,5)	59 (27,8)	0,885**
	Usa	58 (71,6)	95 (72,5)	153 (72,2)	
Uso de drogas ilícitas en los últimos 12 meses*	Nunca usó o ya usó y no usa más	53 (65,4)	77 (58,8)	130 (61,3)	0,334**
	Usa	28 (34,6)	54 (41,2)	82 (38,7)	

*El número de adolescentes, jóvenes y adultos difirió en estas variables, ya que los datos en blanco/ignorados/no aplicables no se tuvieron en cuenta en los análisis;

**Prueba Chi-cuadrado; †Prueba exacta de Fisher;

^+^residuos estandarizados y ajustados por encima de 1,96 indicaron una asociación positiva;

^-^residuos por debajo de -1,96 indicaron una asociación negativa.

Fuente: Elaborado por los autores, 2023.

En cuanto a las relaciones sexuales en los últimos 12 meses, la mayoría (71.2 %) eran HSH y el 48.6 % tenían una pareja estable y ocasional al mismo tiempo. Entre los casos con pareja estable, la mayoría (73,8 %) tenía una relación con “una” pareja estable, mientras que entre los casos que tenían pareja ocasional, la mayoría (83,7 %) se relacionó con “más de una persona en los últimos 12 meses. Al analizar todos los casos, se observó que la mayoría mantenía relaciones solo con hombres (79.7%), practicaba sexo anal receptivo (75.4%), anal insertivo (68.8%), oral receptivo (76.9%) y oral insertivo (76.1%). Los participantes en el estudio informaron que a veces usaban condones (78.3%) y a veces usaban gel lubricante (56.9 %). En cuanto a las parejas sexuales, se observó que el 27.6 % de ellas eran personas que vivían con VIH/ITS, el 14.6 % tenían otras parejas y el 17.6% consumían drogas ilícitas. También se reveló una asociación entre los jóvenes y ser HSH, así como entre los adultos, ser heterosexuales y tener relaciones sexuales con parejas que vivían con VIH/ITS (Tabla 2).

**Tabla 2:** Distribución de jóvenes y adultos con prueba reactiva al VIH (N = 231) según el comportamiento sexual en los últimos 12 meses – Ribeirão Preto, SP, Brasil, 2018 a 2021.

Variable		N = 84* Adolescentes y jóvenes	N = 147* Adultos	Total	Valor p**
		n (%)	n (%)	n (%)	
Grupos poblacionales*	Heterosexual	11/78 (14,1)	37/143 (25,9)	48/221 (21,7)	0,043**
	HSH	62/78 (79,5)	96/144 (66,7)	158/222 (71,2)	0,044**
	Profesional del sexo	8/79 (10,1)	6/143 (4,2)	14/222 (6,3)	0,076†
Tipo de relación	Fija	19 (23,2)	36 (26,5)	55 (25,2)	0,863
	Eventual	22 (26,8)	35 (25,7)	57 (26,1)	
	Fija e eventual	41 (50,0)	65 (47,8)	106 (48,6)	
Número de relaciones fijas en los últimos 12 meses	1	30 (66,7)	60 (77,9)	90 (73,8)	0,395
	2	8 (17,8)	9 (11,7)	17 (13,9)	
	3 o 4	7 (15,6)	8 (10,4)	15 (12,3)	
Número de relaciones eventuales en los últimos 12 meses	1	9 (15,8)	15 (16,5)	24 (16,3)	0,320
	2	5 (8,8)	16 (17,6)	21 (14,3)	
	3–10	23 (40,4)	39 (42,9)	62 (42,2)	
	11–50	13 (22,8)	16 (17,6)	28 (19,0)	
	>50	7 (12,3)	5 (5,5)	12 (8,2)	
Relación sexual en los últimos 12 meses	Solamente hombres	69 (84,1)	104 (77,0)	173 (79,7)	0,107
	Solamente mujeres	5 (6,1)	21 (15,6)	26 (12,0)	
	Hombres y mujeres	8 (9,8)	10 (7,4)	18 (8,3)	
Práctica sexual en los últimos 12 meses	Vaginal	16/79 (20,3)	36/126 (28,6)	52/205 (25,4)	0,183
	Anal receptivo	65/69 (82,3)	88/124 (71,0)	153/203 (75,4)	0,068
	Anal insertivo	54/76 (71,1)	83/123 (67,5)	137/199 (68,8)	0,597
	Oral receptivo	63/77 (81,8)	90/122 (73,8)	153/199 (76,9)	0,190
	Oral insertivo	63/76 (82,9)	87/121 (71,9)	150/197 (76,1)	0,078
Uso de preservativo en los últimos 12 meses	Siempre utiliza preservativo	19 (24,4)	24 (20,0)	43 (21,7)	0,467
	A veces o nunca utiliza preservativo	59 (75,6)	96 (80,0)	155 (78,3)	
Uso de lubricante en los últimos 12 meses	Siempre utiliza lubrificante	27 (42,2)	35 (43,8)	62 (43,1)	0,851
	A veces o nunca utiliza lubrificante	37 (57,8)	45 (56,2)	82 (56,9)	
Vulnerabilidad de la relación en los últimos 12 meses	Vive con VIH/IST	12/78 (15,4)	43/121 (35,5)	55/199 (27,6)	**0,002**
	Múltiples relaciones	9/78 (11,5)	20/121 (16,5)	29/199 (14,6)	0,330
	Usaba drogas ilícitas	13/78 (16,7)	22/121 (18,2)	35/199 (17,6)	0,784

*El número de adolescentes, jóvenes y adultos difirió en todas las variables, ya que los datos en blanco/ignorados/no aplicables no se tuvieron en cuenta en los análisis;

**Prueba Chi-cuadrado.

Fuente: Elaborado por los autores, 2023.

El historial médico de la mayoría de las personas estudiadas reveló relaciones sexuales sin protección (81,8%) y haber realizado alguna prueba del VIH (54,2%) o de sífilis (52,7%) a lo largo de su vida. Entre los resultados de las pruebas serológicas realizadas a jóvenes y adultos, el 29,1% dio positivo en sífilis y el 11,7% había sido diagnosticado con una ITS distinta del VIH en los últimos 12 meses. Se observó una asociación estadísticamente significativa entre las pruebas positivas para el VIH y los jóvenes que declararon haberse realizado más de una prueba de VIH y sífilis en su vida (Tabla 3).

**Tabla 3:** Distribución de jóvenes y adultos con pruebas reactivas al VIH (N = 231) según antecedentes de salud – Ribeirão Preto, SP, Brasil, 2018 a 2021.

Variable		N = 84* Adolescentes y jóvenes	N = 147* Adultos	Total	Valor p**
		n (%)	n (%)	n (%)	
Tipo de exposición*	Relación sexual desprotegida	65/78 (83,3)	115/142 (81,0)	180/220 (81,8)	0,666
	Ruptura de preservativo	14/80 (17,5)	22/142 (15,5)	36/222 (16,2)	0,697
	Relación tuvo o tiene IST	12/78 (15,4)	33/142 (23,2)	45/220 (20,5)	0,167
Primer test en la vida de HIV*	Si	4 (25,0)	18 (56,3)	22 (45,8)	**0,041**
	No	12 (75,0)	14 (43,7)	26 (54,2)	
Primer test en la vida de sífilis*	Si	3 (23,1)	16 (59,3)	19 (47,5)	**0,032**
	No	10 (76,9)	11 (40,7)	21 (52,5)	
Test de sífilis*	Reactivo	25 (32,9)	34 (26,8)	59 (29,1)	0,352
	No reactivo	51 (67,1)	90 (73,2)	144 (70,9)	
Ha sido diagnosticado con una ITS en los últimos 12 meses*	Si	12 (15,0)	13 (9,8)	25 (11,7)	0,251
	No	68 (85,0)	120 (90,2)	188 (88,3)	

*El número de adolescentes, jóvenes y adultos difirió en todas las variables, ya que los datos en blanco/ignorados/no aplicables no se tuvieron en cuenta en los análisis;

**Prueba Chi-cuadrado.

Fuente: Elaborado por los autores, 2023.

## DISCUSIÓN

En el presente estudio, se identificó que la mayoría de los registros analizados correspondían a personas menores de 30 años, lo que evidencia que la infección por el VIH ha afectado a poblaciones en la plenitud de su juventud y de su vida productiva. Las personas más jóvenes tienden a tener un menor nivel educativo y un bajo conocimiento sobre el VIH, además de estar expuestas a diferentes situaciones de vulnerabilidad. En este contexto, son necesarias acciones en salud dirigidas a los adolescentes, con un enfoque en la construcción de conocimientos que se perpetúen hasta la edad adulta^(14)^.

Cabe destacar que se observaron cinco individuos con prueba reactiva al VIH entre 15 y 17 años, lo que demuestra la iniciación precoz de la actividad sexual y la relevancia de los CTA como servicios estratégicos para el diagnóstico del VIH en poblaciones jóvenes. En relación con el grupo de edad identificado, que aún se encuentra dentro del sistema educativo, es urgente implementar actividades innovadoras, basadas en la promoción de la salud sexual y reproductiva y en estrategias de prevención y diagnóstico oportuno mediante enfoques intersectoriales de la Atención Primaria de Salud (APS) y la inclusión de escuelas y otros espacios sociales^(15)^.

Además, los servicios de salud deben garantizar que la información sobre la prevención de las ITS y el VIH se elabore de forma clara y objetiva, abordando aspectos como las medidas profilácticas, la ventana inmunológica en el momento de la prueba y las posibilidades de supresión viral^(16,17)^.

Al analizar las demás características sociodemográficas, se observó que las variables relacionadas con la situación marital, el nivel educativo y la situación laboral reflejaban el perfil esperado entre los grupos estudiados. Así, una parte significativa de los jóvenes eran solteros, estaban estudiando y presentaban un nivel educativo inferior en comparación con los adultos. Se observó, además, que muchos jóvenes estaban empleados, lo que demuestra que la infección por el VIH no constituía un impedimento para el desempeño de actividades laborales entre adultos y jóvenes.

Las demás características sociodemográficas, cuyas asociaciones no tuvieron relación con los grupos estudiados, merecen ser destacadas debido al elevado número de hombres cisgénero y que se declararon de raza blanca entre los que viven con el VIH, lo que refuerza que el reconocimiento de estas vulnerabilidades puede conducir a estrategias más dirigidas a determinados grupos sociales, así como a ser más asertivas en la prevención y el diagnóstico de nuevos casos de VIH^(18)^.

En relación con el consumo de sustancias psicoactivas, alrededor del 70% de las personas estudiadas informaron consumir alcohol y el 40% consumir drogas ilícitas, valores que pueden considerarse elevados. Se entiende que el uso de estas sustancias influye en el uso inconsistente del preservativo y, por lo tanto, aumenta la vulnerabilidad a las ITS y al VIH. Por lo tanto, es esencial que la población comprenda los riesgos que implican las prácticas sexuales bajo la influencia del alcohol y las drogas, construyendo conocimientos que favorezcan comportamientos más seguros^(19)^. La prevención del consumo de alcohol y sustancias psicoactivas debe estar articulada con las políticas públicas de salud, educación y protección social^(19,20)^, garantizando un enfoque amplio del problema en cuestión.

En las variables relacionadas con el comportamiento sexual, ser joven se asoció con los hombres que tienen relaciones sexuales con otros hombres y los adultos con ser heterosexuales. Este hallazgo evidencia la elevada vulnerabilidad del grupo de hombres que tienen relaciones sexuales con hombres, que históricamente es el más afectado por el VIH en Brasil, aunque también se observa un aumento de los casos entre los heterosexuales^(3,21)^.

Otra característica de vulnerabilidad entre los adultos fue la presencia de parejas que vivían con el VIH y otras ITS. En este caso, las estrategias dirigidas a estas personas pueden fomentar un uso más sistemático de lubricantes y preservativos para reducir el riesgo de transmisión de estas infecciones. El número significativo de participantes en el estudio que informaron que a veces o nunca utilizaban estos métodos evidencia la necesidad de iniciativas de salud que promuevan el conocimiento sobre los riesgos de infección por el VIH durante las prácticas sexuales, además del asesoramiento antes y después de la prueba.

En cuanto al comportamiento sexual, muchos de los individuos estudiados mencionaron mantener relaciones esporádicas y múltiples, lo que aumenta la exposición al VIH. Ante esto, es esencial garantizar los derechos a prácticas sexuales más seguras y saludables entre los jóvenes, concienciándolos sobre los riesgos asociados al inicio precoz de las prácticas sexuales y a las relaciones establecidas a lo largo de la vida^(19)^.

Además, una parte significativa de los individuos mantenía relaciones sexuales solo con hombres, lo que demuestra que este grupo puede ser el mayor transmisor del VIH, sobre todo cuando la práctica sexual se realiza por vía anal. Cabe destacar el elevado número de prácticas de sexo oral, que deben realizarse con preservativo, aunque presenten un bajo riesgo de transmisión del VIH en comparación con otras vías sexuales. Durante las consultas de asesoramiento, es importante aclarar los riesgos asociados a las diferentes prácticas sexuales, destacando que la vulnerabilidad a la infección por el VIH varía según la práctica sexual, con mayores posibilidades de transmisión del virus en el sexo anal receptivo, el sexo anal insertivo, el sexo vaginal receptivo, el sexo vaginal insertivo y el sexo oral, respectivamente^(22)^.

En el historial médico, los jóvenes estaban asociados con haber realizado otras pruebas de VIH y sífilis en su vida antes del diagnóstico, lo que indica una posible difusión de las acciones de prueba en esta población. Esto contribuye al logro de los objetivos de la estrategia 95–95–95, que pretende que el 95% de las personas con VIH conozcan su estado serológico, que el 95% de las personas que conocen su estado serológico estén en TARV y que el 95% de las personas que están en TARV tengan la carga viral suprimida^(23)^, lo que las hace indetectables e intransmisibles.

En cuanto al historial de salud, la mayoría de los participantes informaron haber estado expuestos al VIH debido a relaciones sexuales sin protección, lo que también aumenta la vulnerabilidad a otras ITS, como la sífilis, diagnosticada en aproximadamente el 30% de la muestra. Estos datos refuerzan la necesidad de actividades de educación en salud entre la población, lo que permite la adopción de prácticas más seguras como parte de la prevención de estas infecciones, que también debe incluir la distribución de preservativos, gel lubricante y asesoramiento sobre el VIH.

Otro enfoque eficaz en la prevención del VIH es el mandala de la prevención combinada, que debe adaptarse a las realidades y preferencias individuales o colectivas. Esta estrategia incluye diferentes acciones, como la profilaxis postexposición (PEP) y la profilaxis preexposición (PrEP), la prevención de la transmisión vertical, la inmunización contra la hepatitis B y el VPH, la reducción de daños, el diagnóstico y el tratamiento de las ITS y las hepatitis virales, el uso sistemático de preservativos con gel lubricante, tratamiento de todas las personas que viven con el VIH y pruebas periódicas de detección del VIH, las ITS y las hepatitis virales^(24,25)^.

La disponibilidad de estas tecnologías refuerza la importancia de adaptarlas al perfil poblacional e individual de cada región, teniendo en cuenta la realidad y el comportamiento sexual de las personas y sus parejas. En este contexto, cabe destacar también la importancia de ampliar las pruebas rápidas del VIH en espacios extramuros, como escuelas, saunas, fiestas, plazas y otros lugares donde se producen encuentros y prácticas sexuales, favoreciendo el acceso a la prevención y al diagnóstico precoz^(26,27)^.

Con el fin de contribuir a la disminución de la transmisión del VIH, la enfermería desempeña un papel importante en los CTA, ya que su actuación abarca la capacitación del equipo multidisciplinario en cuestiones de prevención del virus y promoción de la salud, la realización de atenciones y asesoramientos individuales y colectivos, además de la solicitud de exámenes de VIH y otras ITS. De este modo, la enfermería debe estar presente en todo el proceso de atención para orientar y aclarar dudas, desde la acogida hasta la consulta posterior^(28)^.

El análisis del perfil y el comportamiento de las personas con resultados positivos en las pruebas del VIH es esencial para orientar las estrategias que reduzcan la transmisión del virus entre poblaciones específicas y en situación de mayor vulnerabilidad. Esta comprensión permite adaptar las medidas de prevención de forma individualizada y colectiva, con un enfoque centrado en la persona, lo que permite que las acciones de promoción de la salud estén alineadas con las políticas públicas, que abordan de manera transversal la prevención de las ITS y el VIH, además de promover la concienciación sobre la reducción de daños, la distribución y el uso sistemático de preservativos, la aplicación de gel lubricante y el fomento de las pruebas serológicas. Más allá de la actuación de los CTA, cabe destacar la importancia de la coordinación de las acciones por parte de la APS, dada la complejidad y la diversidad de los factores asociados a la infección por el VIH entre jóvenes y adultos. La APS debe integrarse con los demás niveles de atención de la RAS, establecienddiscussiono conexiones con políticas y acciones intersectoriales para fortalecer la respuesta a la disminución de la transmisión del VIH.

Entre las limitaciones de este estudio, cabe destacar un posible sesgo de información debido a la falta de validación de la ficha de atención del CTA, lo que puede haber comprometido su adecuación al nivel de conocimiento de los participantes. Además, se señala como segunda limitación el hecho de que su población sea pequeña y restringida a un solo municipio, lo que puede impedir la generalización de los hallazgos y la identificación de las vulnerabilidades que afectan a las personas afectadas por el VIH en Brasil.

## CONCLUSIÓN

En el presente estudio, se observó que la mayoría de las personas incluidas tenían menos de 30 años, eran hombres cisgénero, de raza blanca, con trabajo, que consumían alcohol, utilizaban preservativos y gel lubricante de forma inconsistente, tenían parejas ocasionales y múltiples, y se relacionaban exclusivamente con otros hombres. Entre los jóvenes, se destacó la asociación con el hecho de ser hombres que tienen relaciones sexuales con hombres y haber realizado otras pruebas de VIH y sífilis a lo largo de su vida. Entre los adultos, fue más frecuente la identificación como heterosexuales y la presencia de parejas que vivían con el VIH u otras ITS. Comprender el perfil y el comportamiento de estas personas con pruebas reactivas al VIH es fundamental para respaldar estrategias de salud dirigidas a reducir la transmisión del virus entre poblaciones específicas y en mayor situación de vulnerabilidad.

## DISPONIBILIDAD DE DATOS

Los datos se encuentran disponibles en un repositorio electrónico, accesibles a través del siguiente enlace: https://repositorio.usp.br/item/003225747.
